# Responding to social and symbolic extrafoveal cues: cue shape trumps biological relevance

**DOI:** 10.1007/s00426-015-0733-2

**Published:** 2015-12-26

**Authors:** Frouke Hermens, Markus Bindemann, A. Mike Burton

**Affiliations:** 1 School of Psychology, Brayford Pool, University of Lincoln, LN6 7TS Lincoln, UK; 2 School of Psychology, Keynes College, University of Kent Canterbury, Kent, CT2 7NP UK; 3 Department of Psychology, University of York, York, YO10 5DD UK

**Keywords:** Social cueing, Symbolic cues, Mouse tracking, Eye tracking, Extrafoveal cueing

## Abstract

Social cues presented at visual fixation have been shown to strongly influence an observer’s attention and response selection. Here we ask whether the same holds for cues (initially) presented away from fixation, as cues are commonly perceived in natural vision. In six experiments, we show that extrafoveally presented cues with a distinct outline, such as pointing hands, rotated heads, and arrow cues result in strong cueing of responses (either to the cue itself, or a cued object). In contrast, cues without a clear outline, such as gazing eyes and direction words exert much weaker effects on participants’ responses to a target cue. We also show that distraction effects on response times are relatively weak, but that strong interference effects can be obtained by measuring mouse trajectories. Eye tracking suggests that gaze cues are slower to respond to because their direction cannot easily be perceived in extrafoveal vision. Together, these data suggest that the strength of an extrafoveal cue is determined by the shape of the cue outline, rather than its biological relevance (i.e., whether the cue is provided by another human being), and that this shape effect is due to how easily the direction of a cue can be perceived in extrafoveal vision.

## Introduction

Many studies have demonstrated strong effects of gaze cues on the attention and eye movements of observers (Driver et al., 1999; Friesen & Kingstone, 2003a, b; Frischen, Bayliss & Tipper, 2007a; Frischen, Smilek, Eastwood & Tipper, 2007b; Hermens & Walker, 2010a; Itier, Villate & Ryan, 2007; Kuhn & Benson, 2007; Kuhn & Kingstone, 2009; Kuhn, Tatler & Cole, 2009; Langton, Watt & Bruce, 2000; Nummenmaa & Hietanen, 2006; Quadflieg, Mason & Macrae, 2004; Ristic, Wright & Kingstone, 2007; Tatler & Kuhn, 2007). In a typical setup, participants are asked to ignore a centrally presented gaze cue and to respond to a peripherally presented target. For cue–target intervals up to around 2 seconds, responses to targets that are gazed at by a face tend to be faster and more accurate, even when the cue is known to be unpredictive or even counterpredictive of the location of the target (Driver et al., 1999). Results such as these have led to speculations about the existence of eye direction detectors (Baron-Cohen, 1995), with a special role of the dark pupil on the light sclera (Ricciardelli, Baylis & Driver, 2000), specialized brain networks for the processing of gaze (Grosbras, Laird & Paus, 2005; Hietanen, Nummenmaa, Nyman, Parkkola & Hämäläinen, 2006; Hoffman & Haxby, 2000), and deficits of social attention in autism spectrum disorders (Leekam, Hunnisett & Moore, 1998; Senju, Tojo, Dairoku & Hasegawa, 2004).

The majority of these studies, however, have been restricted to cues presented at fixation, and one may ask whether this paradigm accurately reflects the effects of social cues in day-to-day vision. In natural vision, observers do not always immediately fixate the cue. Instead, the observer’s gaze can start elsewhere in a scene, and an eye movement is required to first fixate the cue (face or eyes). Only after this eye movement is the situation of typical gaze cueing experiments achieved. Initially therefore, cues can be perceived outside the fovea (the central two degrees of the visual field, Rayner 1998). To extrapolate the results from past studies using the standard gaze cueing paradigm (with gaze cues presented in isolation and fixation) to natural vision, it is, therefore, important to understand the influence of different types of cues (initially) presented outside fixation to ensure that the importance of gaze cues to attention extends beyond laboratory situations.

**Fig. 1:**
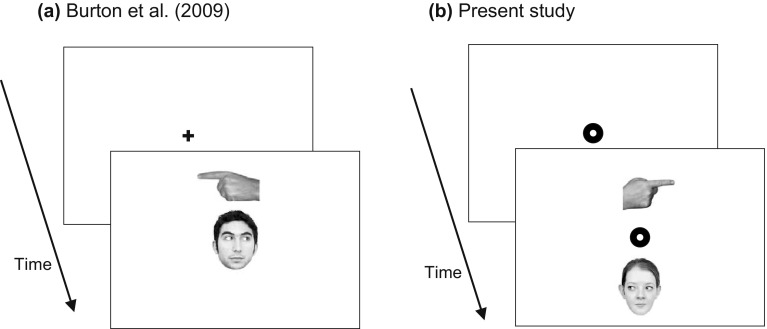
**a** Sequence from Burton et al. (2009) used to examine the influence of extrafoveally presented distractor cues on responses to the centrally presented target. **b** Sequence in the present study, in which both the target and distractor are (initially) shown in extrafoveal vision

While a large number of studies examined the influence of social cues presented at fixation, only a limited number of studies have examined social cues away from fixation (Burton, Bindemann, Langton, Schweinberger & Jenkins, 2009; Langton & Bruce, 2000; Nummenmaa & Hietanen, 2009). To examine the perception of extrafoveally presented social cues, Burton et al. (2009) used a target–distractor paradigm. In a series of experiments, they measured the facilitation or inhibition of responses to centrally presented cues by extrafoveally presented gaze (a face with an averted gaze), eyes-only (the eye region of the face), pointing hand, and rotated head cues. On a typical trial, a target (e.g., a face with its eyes averted) was presented together with a distractor (e.g., a pair of averted eyes or a pointing hand) above or below fixation (Fig. 1a). The experiments were specifically designed to examine the influence of extrafoveally presented cues on responses to another cue (i.e., interference). Because all cues used in the study were of biological relevance, no differences between the cues would be expected if biological relevance would be the determining factor for cueing. Several conclusions could be drawn from the results. First, extrafoveally presented faces or pairs of eyes (each with an averted gaze) did not interfere with either averted gaze targets or pointing hand targets. In contrast, pointing hand distractors significantly interfered with both averted gaze and pointing hand targets. Second, increasing the size of the distractors outside the fovea did not change the results, meaning that retinal size cannot account for the results. Third, reducing the strength of the target (by making it more difficult to detect its orientation) did not increase the effects of extrafoveal gaze distractors. Fourth, restricting the gaze cue to a pair of eyes, reducing possible crowding effects (Levi, 2008; Whitney & Levi, 2011), did not increase distraction from gaze in extrafoveal vision. Finally, rotated heads significantly interfered with responses to averted gaze targets. These differences between the various cues suggest that factors other than biological relevance are at work.

The experiments by Burton et al. (2009) suggest that eye-gaze cues only weakly influence responses to a central target, but it is unclear whether it is attention that is influenced. Instead, the target–distractor paradigm might probe into another stage of processing, such as response preparation. Moreover, only the distractor was presented in extrafoveal vision, which may have given the target an unfair advantage in processing. These possible issues were addressed by Nummenmaa and Hietanen (2009) who asked participants to remain fixated on the center of the screen while two possibly conflicting cues were presented in extrafoveal vision. Participants were instructed to attend to one of the two stimuli until the appearance of the response target either in the direction of the attended cue, or in the opposite direction. In such a setup, gaze and arrow cues showed similar extrafoveal cueing (as the attended stimulus) and distraction effects (as the unattended stimulus).

The studies by Burton et al. (2009) and Nummenmaa and Hietanen (2009) both prevented eye movements to the stimuli away from fixation, thereby ensuring that the influence of the stimuli was purely from extrafoveal vision. In natural vision, however, observers make eye movements to relevant stimuli, and it is, therefore, important to also examine the situation in which stimuli are initially presented away from fixation after which they are foveated. Langton et al. (2000) instead presented stimuli until the observer’s response and did not prevent eye movement to different aspects of the stimuli. Interestingly, this study provided similar effects of the extrafoveally presented pointing, head gaze, and arrow cues (provided by a single actor) even when eye movements were allowed.

Results from these experiments are sometimes contradictory (e.g., extrafoveal gaze effects in Nummenmaa & Hietanen, 2009, but not in Burton et al., 2009), but they seem to suggest that cues effective at fixation are not necessarily effective away from fixation. At fixation, social cues (in particular eye gaze) provide strong cueing, while away from fixation, cues whose shape can easily be distinguished (rotated heads, pointing hands, arrows) appear to have similar or stronger influences. A possible reason is that only for cues with a distinct shape, the direction of the cue can already be seen in extrafoveal vision, while for cues with a less distinct shape outline (such as gaze cues), an eye movement is first required to the cue. With the present experiments, we aim to test these hypotheses. Our approach is to test multiple extrafoveally presented cues directly against each other within the same experiment (past studies were restricted to comparisons of pairs of cues), so that a ranking of cue strengths is obtained. Target and distractor cues are presented in such a way that both are (initially) away from fixation (Fig. 1b; similar to the situation in Langton et al., 2000), but in all but one of our experiments, we allow participants to make eye movements to mimic the natural situation in which cues are normally perceived. Our hypothesis is that if biological relevance (i.e., being provided by a human being) is the determining factor for the cue’s strength, gaze cues (either provided by eye-gaze shifts or head turns) or pointing gestures should influence responses more strongly (either as a target or a distractor), but there should be no differences between the various biological cues. Alternatively, if the shape of the cue determines its strength, cues with a distinct shape (pointing hands, arrows, and to a weaker extent, rotated heads) should have the strongest influence on observers (again, either as a target, or a distractor), independent of whether the cue is provided by a human being. In the first experiment, we compare different social cues (those from Burton et al., 2009) in an interference task in which participants respond to the direction of a predefined target, while ignoring a distractor stimulus (one of the other cues). In the second experiment, a social (gaze) cue is compared against two symbolic cues (arrow and direction word) and a sudden onset, providing a range of social and symbolic cues to test the above hypotheses.

In addition to our main question, our experiments aim to answer several methodological questions. In our third experiment, we determine whether the ranking of the cues depends on whether responses to the cue itself are measured, or whether responses to a cued object are recorded. Responses to the cues are faster and easier to produce than responses to cued objects. If we can demonstrate that the ranking of cues is independent of whether the cue or a cued object is responded to, this will facilitate research into establishing what exactly determines the strength of a direction cue. It will also establish whether discrepancies in earlier results (Burton et al., 2009; Nummenmaa & Hietanen, 2009) were due to this aspect of the paradigm (as this was one of the aspects on which the studies differed). In our fourth experiment, we examine whether stronger influences of distractor stimuli can be obtained by relying on a different response measure, by asking participants to move the mouse cursor to one of four response boxes, measuring the curvature of the mouse trajectories. Mouse trajectories have been used successfully to measure the time-course of deliberation between responses (Freeman, Dale & Farmer, 2011), requiring fewer repeated trials than response times measures (e.g., using event history analysis, Panis & Hermens 2014). Our fourth experiment will establish whether mouse trajectories provide an efficient measure of interference from conflicting cues of direction. The first four experiments together also aim to determine whether cues that can be responded to more easily also provide stronger interference if they have to be ignored (i.e., whether the ranking of cues depends on whether they serve as a target or a distractor).

Finally, we aim to probe into the origin of the differences between the various cues. In the fifth experiment, we therefore determine whether eye movements are more often made to cues that have longer response times, to determine whether these longer response times can, in part, be understood from the time needed to move the eyes to the cue. Finally, in the sixth experiment, we compare cues when participants are not allowed to look at them, to determine whether cues that are responded to more slowly and produce stronger interference are those cues that can be more easily discriminated in extrafoveal vision.

In our experiments, we stay close to the original experiments by Burton et al. (2009) and make relatively small changes in going from one experiment to another. With this approach, we aim to reveal consistent effects across a series of experiments with minor differences, to avoid placing strong focus on effects that may reach significance only once. Making small changes also allows for determining what change in condition leads to a change in results. Some of the changes that we are making involve differences between the paradigms by Burton et al. (2009) and Nummenmaa and Hietanen (2009), such as the target stimulus for the response (the cue or a cued object), which may shed some light on the inconsistencies in past results.

## Experiment 1

Experiment 1 compared the four types of cues (faces, eyes, heads, and hands) introduced by Burton et al. (2009) in a single experiment, so that a ranking of their influence can be determined. On each trial, two of the four cues were presented above and below fixation and participants were asked to report the direction of the predefined target cue (e.g., “in this block, always respond to the hand stimulus”) while ignoring the other stimulus. If biological relevance of the cues is the determining factor for the cue’s strength, response times to the four different cues as targets should not differ significantly (because all cues are social cues). As distractors, the cues should facilitate (congruent distractors) and impair (incongruent distractors) responses to the target similarly. In contrast, if the shape of the cue determines the strength of a cue, we expect the hands, and to a lesser extent, the rotated heads, to yield the fastest response times as a target, and the strongest interference as distractors. Because Experiment 1 asked participants to respond to the cue, it is expected that any difference between cues reflects response preparation.

### Methods

#### Participants

Twenty psychology students from the University of Aberdeen participated as part of a first year course. They all provided written consent for participation in the experiment that was approved by the local ethics committee.

#### Apparatus

Stimuli were presented by means of a dual-core Dell Pentium PC onto a 19 inch Dell LCD screen, viewed at a distance of approximately 70 cm, using the OpenSesame software package (Mathôt, Schreij & Theeuwes, 2012). Responses were collected using a standard USB keyboard.

**Fig. 2:**
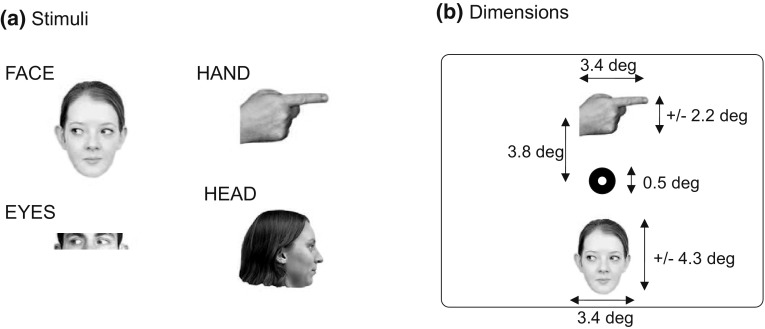
**a** Examples of stimuli in Experiment 1. **b** An example array with stimulus dimensions

#### Stimuli

Stimuli were adopted from Burton et al. (2009) (illustrated in Fig. 2a), and consisted of pictures of eyes gazing left or right within a face (FACE), only the eye-region gazing left or right (EYES), hands pointing left or right (HAND), and heads rotated left and right (HEAD). These pictures were from three male and three female actors, whose identity was unknown to the participants. Pictures were scaled down to a width of 4.2 cm (3.4° of visual angle at the 70 cm viewing distance used), but could vary in their vertical size (Fig. 2b). Their distance to the fixation point (itself measuring 0.5° in diameter) was 3.8°, so that no stimulus spatially overlapped with the fixation point. Luminance measures, taken using an LX-101 Lux meter, indicated an approximate luminance of the areas occupied by the FACE stimuli of around 100 Lux, of the EYES of around 120 Lux, of the HANDs of around 105 Lux, and of the HEADs of around 25 Lux, against a background of 146 Lux.

#### Design

An incomplete four (targets) by five (distractors) by two (congruency) design was used. Combinations of a target (FACE, EYES, HAND and HEAD) and a distractor (FACE, EYES, HAND, HEAD, and no distractor) were presented on each trial, with congruent or incongruent pairings of the directions of the target and the distractor. For each target, there were 12 trials of each distractor in the congruent condition, 12 trials in the incongruent condition, and 12 no-distractor trials. Targets were presented in separate blocks, allowing for an instruction on the target to be given before each block. Distractors, target direction and congruency were randomly intermixed within each block. The location of the target (above or below fixation) was randomly selected on each trial. The duration of the fixation point before stimulus onset was randomly set to be between 600 and 1200 ms. The order of the blocks was counterbalanced across participants to counteract effects of fatigue and practice in the overall data. Each block started with three practice trials, randomly selected from the trials of the upcoming block.

#### Procedure

Participants were instructed to respond to the direction of a target cue by pressing the corresponding button on the keyboard, while ignoring the second cue on the screen. The target cue was announced before the start of each block (e.g., “In this block, respond to the face stimulus”), together with some example pictures, followed by three practice trials with feedback. Trials started with a fixation point presented for 600ms to 1200ms. Two cues then appeared above and below fixation (Fig. 1b) until participants provided their response. The screen was then cleared and feedback on the accuracy of the response was provided (“Correct!” in green or “Incorrect” in red). Within each block, feedback on the average response time and accuracy was provided after the 32nd and 62nd trial, also allowing participants to take a short break. Each block contained 84 trials, and the experiment took about 25 minutes.

#### Data analysis

The focus of our analyses will be on response times, as error rates were generally low. As in Burton et al. (2009), median response times were computed for each participant, reducing the influence of outliers without the need of data filtering. Response times for targets in the absence of distractors (as a measure of the effects of the targets on their own) were analyzed using univariate repeated measures ANOVAs (Greenhouse–Geisser corrected where appropriate), and in case of significant differences, followed by Bonferroni-corrected paired samples *t* tests. Because of the incomplete design (no trials with the same stimulus type as target and distractor were used), separate repeated measures ANOVAs (for each target) were needed to test for the joint effects of distractor congruency and distractor type. These ANOVAs often led to a significant interaction between the two factors, creating the need for post hoc tests. To avoid crowding the results sections with these statistics, we only report the post hoc test results in the form of Bonferroni-corrected paired samples *t* tests.

### Results

**Fig. 3:**
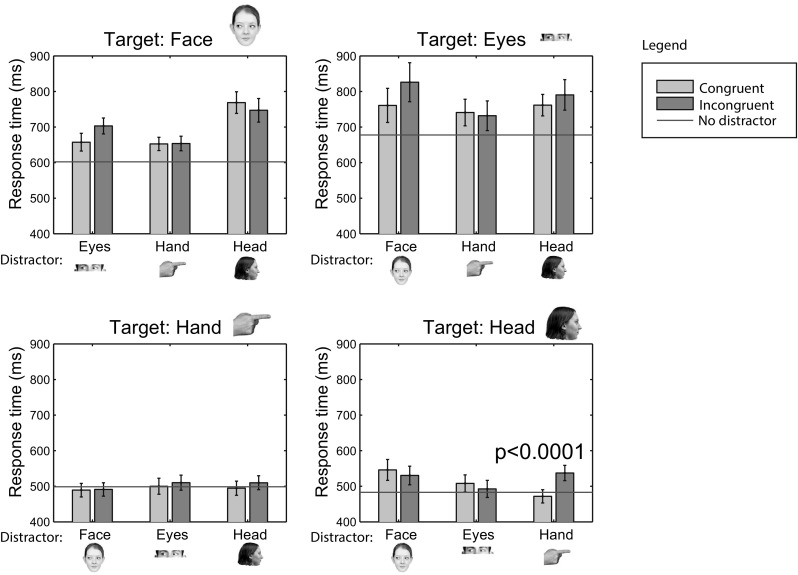
Median response times in Experiment 1. *Green bars* denote congruent conditions, *red bars* incongruent conditions, and horizontal *blue lines* the conditions in which the target was presented without a distractor. Each *subplot* shows the data for the target indicated in its title. Distractors are shown on the *horizontal axis*. *Error bars* indicate the standard error of the mean across participants. *p* values indicate significant congruent–incongruent differences. Color images can be found in the online version (color figure online)

No participants were found with excessive error rates (the largest overall error rate was 6.5 %, with an average of 2.3 % across participants; standard deviation of 1.8). Figure 3 shows the median response times (averaged across participants) in Experiment 1. The overall pattern of results suggests faster response times for target HANDs and HEADs, compared to EYES and FACEs. When no distractors are used (horizontal lines in Fig. 3), response times differ significantly across targets [*F*(1.96, 37.3) = 54.3, *p* < 0.001, $\eta _\mathrm{p}^2$ = 0.74]. Bonferroni-corrected paired samples *t* tests showed significant differences between all pairs of cues (all *p* < 0.0036), except between HAND and HEAD cues (only the significant values are shown in the data plots; the full set of comparisons will be available as online materials). The influence of the different cues as distractors was examined by comparing congruent and incongruent response times and error rates using Bonferroni-corrected paired samples *t* tests (critical *p* value corrected to 0.0042 for 12 comparisons). As shown by the *p* value above the bars in Fig. 3, the only significant distraction effect was obtained from HANDs on HEAD cues. The results, therefore, show large differences between cues as targets (in the absence or presence of distractors), but smaller or no differences as distractors. Strongest influences were found from cues with a clear shape (HAND and HEAD cues).

## Experiment 2

Experiment 1 compared different social cues. An important debate in the literature, however, is whether social cues are special in the sense that they lead to faster response selection and attention shifts than symbolic cues (typically arrow cues are considered, but direction words have also been used). In particular, it has been questioned whether social cues have similar effects on attention and response selection as sudden onsets, which are assumed to result in exogenous shifts of attention, compared to symbolic cues, which are thought to result in endogenous shifts of attention (Müller & Rabbitt, 1989). While Nummenmaa and Hietanen (2009) and Langton and Bruce (2000) compared social (eye gaze, turned heads, pointing hands) and symbolic cues (arrow signs), no comparison was made with sudden onsets. Experiment 2, therefore, compares extrafoveally presented social (eye gaze) and symbolic (arrow and direction word) cues against sudden onsets (occurring at one of the two response locations, rather than above or below fixation). If biological relevance determines the strength of a direction cue, fastest responses should be expected to the gaze cues (as a target) and strongest interference from these cues (as a distractor). In contrast, if shape determines the cue’s strength, faster responses and strongest interference should be found for the arrows, followed by the gaze and word cues (whose outlines do not have such a distinct shape). If gaze cues produce exogenous responses, their influence on response times should be similar to that of the sudden onsets.

### Methods

**Fig. 4:**
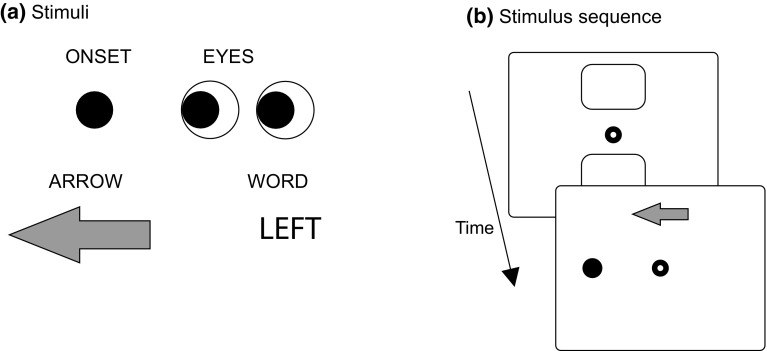
**a** Stimuli used in Experiment 2 (left-response stimuli). **b** Stimulus sequence in Experiment 2. Place-holders were replaced by cues or an empty stimulus (when an onset target or distractor was presented left or right of fixation)

Experiment 2 applied the same method as Experiment 1, and differed only in the stimuli applied. Twenty-five participants (aged between 19 and 25 years) took part, but data from one participant had to be removed due to an issue with data storage in one of the blocks, leaving data for 24 participants (10 male). The stimuli are illustrated in Fig. 4a. A solid black dot (1.6° of visual angle in diameter) served as the ONSET (surface area luminance of 43 Lux). The EYES stimulus, measuring 5.9° by 2.6° in width and height (47 Lux), was created by two circles and two black discs. The ARROW, measuring 6.2° by 2.6° in width and height (65 Lux), was taken from the standard set of Corel Draw, using a gray fill-color. Finally, the WORD stimuli, measuring 5.7 by 1.6 degrees (96 Lux), were shown in capital letters (Arial, 48 points, boldface font). The onset was presented 12.4° left or right from the center of the display (placing the stimulus in the left or right side of the display, sufficiently far from fixation to produce a peripheral onset). The other cues were presented 5.2° above or below fixation. Place-holders (measuring 21.0° by 6.9°) in the form of rectangle outlines, were used before the symbolic and social cues (Fig. 4b) to avoid strong transients, distinguishing the social and symbolic cues from the onset cue, where a unique onset occurred at one of the two response locations. In the no-distractor condition a string of four Xs was used (“XXXX”) in the same font as the word stimuli. All stimuli were presented on a white background (146 Lux). As in Experiment 1, participants were asked to respond to the direction (ARROW, EYES, WORD) or location (ONSET) of the stimuli (left or right). Each block contained 84 trials, with 12 no-distractor trials and 72 trials of congruent and incongruent distractor conditions. As in Experiment 1, stimuli equally often required left and right responses. The target was blocked and an instruction was given before each block indicating which stimulus to respond to. The order of the blocks was randomized across participants so that each order was used once.

### Results

**Fig. 5:**
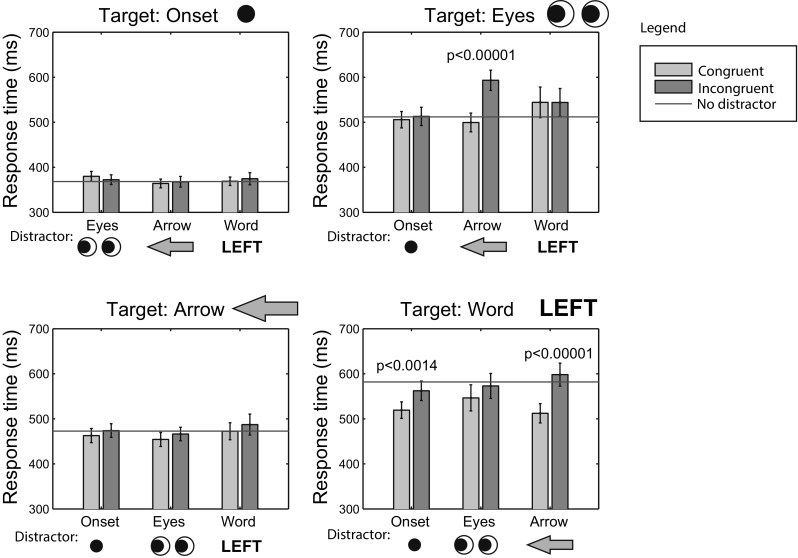
Median response times averaged across participants from Experiment 2. *Green bars* represent congruent trials, *red bars* incongruent trials. The *horizontal lines* indicate the no-distractor condition. *Error bars* show the standard error of the mean (color figure online)

Figure 5 shows the response times in Experiment 2. Response times without distractors (horizontal lines in the different subplots) differed significantly across targets [*F*(1.79, 41.1) = 47.7, *p* < 0.0001, $\eta _\mathrm{p}^2$ = 0.68]. Bonferroni-corrected pairwise comparisons demonstrated significant differences between each of the targets (all *p* values <0.0067), meaning that onsets were responded to fastest, followed by arrows, eyes, and words. Adjacent bars in Fig. 5 show the congruent and incongruent trial response times for each of the possible response targets and distractor items. *p* values in these plots show the significant differences (after Bonferroni correction), showing that incongruent ARROWs significantly slow down responses to EYES, and that response times to WORDs are significantly influenced by the congruency of the ONSET and ARROW distractors.

These results suggest that the central cues with a clear outline (ARROW) influence responses more strongly than cues without such a clear outline shape (EYES, WORD). All central cues (ARROW, EYES, and WORD) were responded to more slowly than sudden onsets, arguing against exogenous influences of the central cues. The faster responses to peripheral onsets cannot be explained from the distance to fixation (the onsets were further from fixation than the central cues), but could relate to the cue being presented at one of the two response locations (resulting in a congruency between stimulus location and response key location), or could be due to the absence of a place-holder before the onset of the stimulus. Interestingly, the ONSETs did not result in significant interference with responses to the EYES, in contrast to the ARROWs. This may be related to the longer distance between the ONSETs and the EYES than between the ARROWs and the EYES.

Another possible confounding factor in the results may be the number of elements that made up the cues. The EYES and WORD cues consisted of multiple elements, while the ARROW cue consisted of a single closed contour only. The elements of the EYES and WORD cues, however, were presented in close proximity, known to be a strong cue for perceptual grouping, and it can, therefore, be reasonably assumed that the elements were perceptually grouped. Another factor may be luminance, which the present experiments did not control for. However, earlier work measuring saccade trajectories for peripheral onset distractors, did not suggest an influence of the luminance or size of the distractor (Hermens & Walker, 2010b).

## Experiment 3

The experiments so far have examined how fast participants can respond to the direction of different cues. One may argue that any target (with or without a distractor) or distractor (congruency) effects in such a paradigm may reflect response preparation rather than the cueing of attention (with the direction cue triggering a response in the cued direction rather than shifting the observer’s attention in the direction indicated by the cue). Experiment 3 investigated whether the same ranking of the direction cues is obtained if the task no longer is to respond to the cue itself, but instead to a cued object (using the stimuli of Experiment 1). If response preparation and cueing of attention rely on similar aspects of the stimuli indicating direction, the same ranking is expected to be achieved for responses to a cued object (Experiment 3) as for responses to the cues themselves (Experiment 1). In contrast, if response preparation and attention cueing depend on different aspects of the cues (e.g., their biological relevance rather than the shape of the cue), a different ranking is expected.

### Methods

**Fig. 6:**
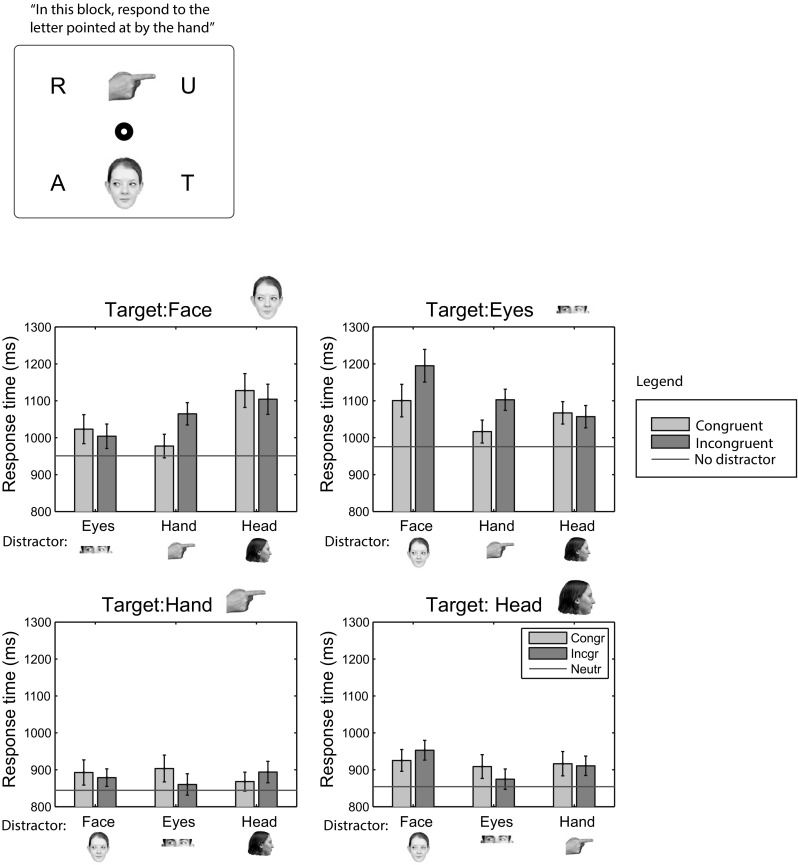
Experiment 3 was identical to Experiment 1, except for the inclusion of cued objects that participants were asked to respond to. The *top* of the figure provides an illustration of a stimulus display and the different conditions. Participants were asked to respond to the *letter being pointed* or looked at by the cue indicated at the beginning of the block (the target cue). For example, if the instruction was to “In this block, always respond to the *letter* indicated by the hand”, and the hand was pointing at the letter “U”, the response required from the participant was an “up” key press (“U” or “up” and “D” for “down”). The *bottom* of the figure shows the median response times to identify the *letter* pointed at or looked at by the target cue, averaged across participants. *Green bars* represent congruent trials, *red bars* incongruent trials. The *horizontal lines* indicate the no-distractor condition. *Error bars* show the standard error of the mean (color figure online)

Experiment 3 was identical to Experiment 1, except for the inclusion of cued objects (letters) that participants were asked to respond to. Twenty-one first and second year students (four male, aged between 16 and 25 years) took part in the experiment in return for course credit. The stimulus layout of Experiment 1 was adjusted to include four letters on each trial (Fig. 6), one in each possible direction and position of the target cue (the cue that was named as the target at the beginning of the block). To allow for a binary response (selection of one of two response keys), the letter that was pointed or looked at by the target cue (as indicated at the start of the block) was selected from the letters “U” (requiring an “up” response) and a “D” (requiring a “down” response). Three letters were placed at the three other locations to increase the probability that participants would use the cues to find the target. These three letters were chosen randomly from the set: “P”, “R”, “Z”, “E”, “B”, “M”, “N”, “S”, and “A” (three different letters on each trial). As in Experiment 1, participants completed four blocks of 84 trials, each with a different target cue (FACE, EYES, HAND and HEAD), named at the beginning of each trial. Twelve of these trials were no-distractor trials (equal numbers of left and right cue trials), and the remaining 72 trials had equal numbers of congruent and incongruent and left and right target trials. The position of the target cue (above or below fixation) was chosen at random for each trial. The experiment took approximately 25 min to complete.

### Results

Horizontal lines in Fig. 6 show the response times to the letters in the absence of distractor stimuli, suggesting faster responses to letters pointed and looked at by HAND and HEAD cues than FACE and EYES cues. A repeated measures ANOVA confirmed the significant differences in distractor-absent response times across the different cues [*F*(3,60) = 15.01, *p* < 0.001, $\eta _\mathrm{p}^2$ = 0.43]. Pairwise comparisons showed significant differences between all cues (*p* < 0.001), except between the FACE and EYES cues and between the HAND and HEAD cues. Effects of the different stimuli as distractors (to the target cue) were weak and Bonferroni-corrected pairwise comparisons did not reveal any significant differences between congruent and incongruent trials. The same ranking of the different cues was obtained as in Experiment 1 (HAND and HEAD cues, stronger than FACE and EYES cues), although the significant difference between FACE and EYES cues was not reproduced in Experiment 3, possibly to due slower overall response times in Experiment 3, allowing for a larger variability in the measurements. The almost identical ranking to Experiment 1 suggests that the strength of the cues does not critically depend on whether participants respond to the direction of the cue, or to cued objects.

## Experiment 4

Experiments 1–3 produced clear rankings of the cues with respect to response times to the cues as targets. The effects of the cues as distractors, however, were weak, and not always consistent. A possible reason could be that the experiments made use of response times, which can be variable across and within participants, and may have limited the statistical power to detect distractor influences. Previous studies have suggested mouse tracking as a viable method to evaluate response conflicts (Freeman & Ambady, 2010; Freeman et al., 2011). In Experiment 4 we, therefore, examine whether mouse tracking may be able to reveal distractor effects more easily than response times. If mouse trajectories provide a sensitive measure of response conflict between the target and distractor stimulus, clear differences between congruent and incongruent trials should be found.

### Methods

Twenty-five students (13 female, average age: 21.5 years, 3 left-handed) took part in the experiment without receiving reimbursement. All reported normal or corrected-to-normal vision and provided written consent for their participation in the study that was approved by the local ethics committee. The experiment was run on the same type (Dell dual core and Dell 19 inch flat screen monitor) of computer setup as in Experiment 1. A standard USB laser mouse (Dell K251D), with the pointer speed set at medium, was used for data collection.

**Fig. 7:**
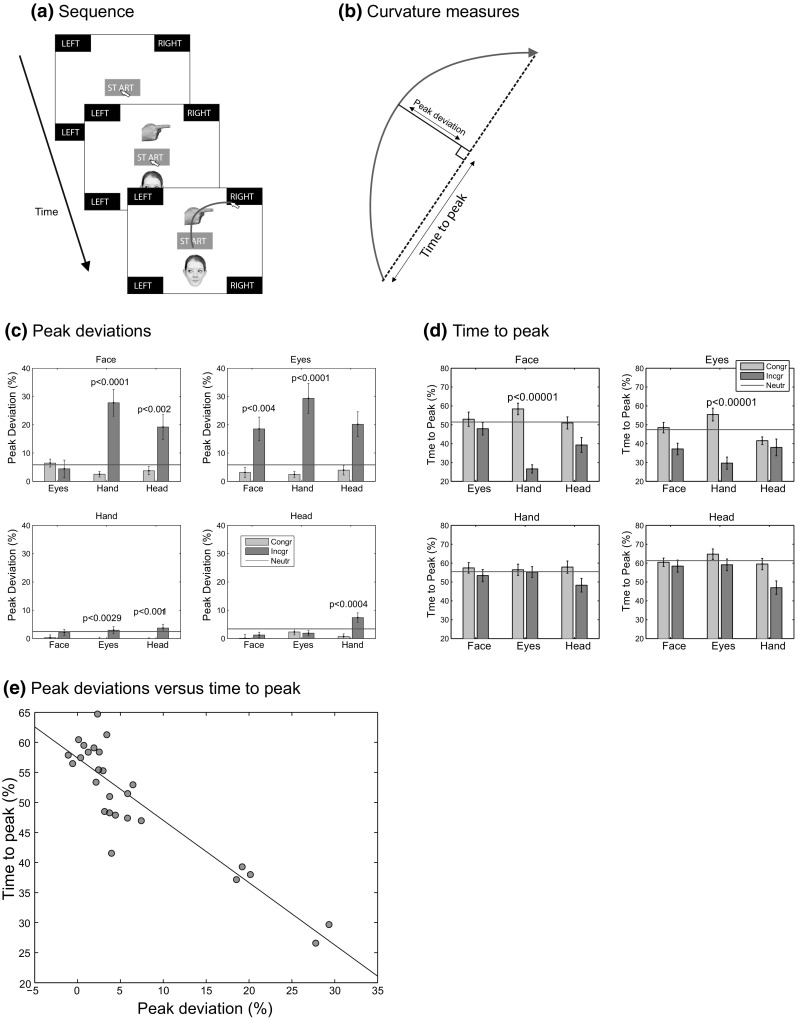
Stimulus sequence, data analysis, and results of Experiment 4. **a** After participants clicked on the START button, they saw two cues, and had to move the mouse as quickly as possible to the button indicated by the target (in this example the hand; indicated at the start of the block) corresponding to the vertical location of the target (*top*, *bottom*). If they did not start their mouse movement within 700 ms, they received a message to start their mouse movement more quickly, and if they clicked on the wrong button a *red X* appeared in the middle of the screen. Note: Images are not to scale. **b** Measurement of curvature of the trajectory, taking the peak deviation as a percentage of the straight-path length. The time-to-peak is also defined as a percentage of the straight-path length. **c** Peak deviation as a percentage of straight-path length. **d** Time to peak as a percentage of the straight-path length. **e** Scatterplot between peak deviation and time to peak. Average data were computed on the basis of correct responses only and reflect median values per participant to reduce the influence of outlier values. *Green* *bars* indicate congruent trials, *red* *bars* incongruent trials. *Horizontal* (*blue*) *lines* indicate no-distractor trials. *p* values indicated in the graphs show those comparisons surviving a 12 comparison Bonferroni correction. *Error bars* show the standard error of the mean (color figure online)

The cues of Experiment 1 were used. The display consisted of a START box (measuring 3.3° by 1.6° of visual angle at the viewing distance of 70 cm), four response boxes (each measuring 4.4° by 3.9°) with the labels ‘LEFT’ and ‘RIGHT’, and the standard Windows 7 arrow cursor to indicate the mouse position (see Fig. 7a, images not to scale). After a mouseclick on the start button, cues were presented above and below the start button at approximately 3.5° of visual angle from the center of the screen (center of the image to the center of the screen). If participants did not start their mouse movement within 700 ms after clicking on ‘START’ the message ‘Please start moving earlier on even if you are not fully certain of a response yet!’ was shown in a pop-up window. If they clicked on the incorrect response box, a red cross was shown in the middle of the screen.

Participants performed four blocks of 84 trials (12 without a distractor, 24 trials with of the three non-target distractors; equal numbers of congruent and incongruent trials and left cue and right cue trials), each of which used one cue (FACE, EYES, HAND, HEAD) as the target. The order of the blocks was varied across participants, so that each possible order was used once (and one order was repeated for the 25th participant). To present both images in Mousetracker an offline Matlab script combined the two images into one, randomly assigning the target to the top or bottom position (and the distractor to the other position). Depending on where the target appeared (above or below fixation), participants had to click the top or bottom response box associated with the direction of the cue (left or right).

Because times to initiate and complete the response showed a similar pattern of results as the manual response times in Experiment 1 (faster responses to HANDs and HEADs than to EYES and FACEs), only the results for the mouse trajectories data will be presented. The trajectories were analyzed for (1) the amplitude of the largest deviation of the path with respect to a straight line between start and response button (Fig. 7b) and (2) the moment in the trajectory at which the maximum deviation occurred (as a measure of the time-course of interference). Both measures are expressed as a percentage of the length of the straight path (e.g., Nummenmaa & Hietanen, 2006). Only correct responses were analyzed. Repeated measures ANOVAs with Greenhouse–Geisser corrections where appropriate and Bonferroni-corrected paired *t* tests were used for statistical comparisons.

### Results

The results from Experiment 4 are shown in Fig. 7c–e. Baseline curvature (without distractors; horizontal lines in Fig. 7c) did not differ significantly across the four cues [*F*(2.20,52.7) = 2.62, *p* = 0.077, $\eta _\mathrm{p}^2$ = 0.098], suggesting that mouse trajectories are not sensitive to the cue that was responded to. Mouse trajectories, however, strongly varied with the distractor. The interference effects are reflected by significant differences between the congruent and incongruent trials (green and red adjacent bars in Fig. 7c). Pairwise comparisons between congruent and incongruent trials revealed significant interference effects of HANDs and HEADs on FACEs, of FACEs and HANDs on EYES, of EYES and HEADs on HANDs, and of HANDs on HEADs. Time to peak shows a similar pattern of results (Fig. 7d), but in contrast to peak deviation, a baseline difference (no distractor trials) was found [*F*(3,72) = 3.54, *p* < 0.019, $\eta _\mathrm{p}^2$ = 0.128]. Paired comparisons showed that this difference was due to a significant difference between EYES and HEADs [*t*(24) = 3.28, *p* = 0.003]. Comparisons between congruent and incongruent conditions showed distractor interference from HANDs on FACEs and from HANDs on EYES (Fig. 7d). To examine whether larger peak deviations are associated with earlier peak times, Fig. 7e plots the two measures against one another, revealing a significant negative correlation (*r* = −0.90, *p* < 0.0001).

### Discussion

Whereas response times (Experiment 1) revealed differences between cues as targets, no such effects were found for mouse trajectories (Experiment 4). Instead, mouse trajectories revealed strong interference effects (Experiment 4) that could not be consistently observed with response times (Experiment 1). The results, therefore, suggest complementary roles for response times and mouse trajectories in ranking cues as response targets and distractors. Importantly, cues that were responded to quickly as a target (HANDs and HEADs) also demonstrated the strongest interference effects using mouse trajectories, suggesting a common underlying mechanism. Peak deviations and time-to-peak showed a significant correlation, casting doubt on the assumption that one measures strength of interference (peak deviation) and the other the time of interference (time-to-peak). Comparisons of mouse trajectories to the no-distractor conditions show that the influence of distractor cues is mostly restricted to interference (from incongruent distractors), while facilitation (by congruent cues) does not seem to occur. Floor effects, however, may play a role, with baselines close to zero leaving little room for congruent cues to make a difference.

The results of Experiment 4 indicate that interference effects that were difficult to detect using response times, can be reliably detected using mouse trajectories. At this point, it is difficult to tell why mouse trajectories provide stronger interference effects than response times. Reviews of the mouse tracking paradigm (Freeman et al., 2011; Hehman, Stolier & Freeman, 2015) have suggested the tight coupling of neural activity of neurons in the motor cortex with ongoing decisions (Cisek & Kalaska, 2005) as a possible reason why mouse trajectories provide a strong measure of response conflict, but it is unclear why such effects would be limited to mouse trajectories and do not extend to response times.

While Experiments 1 to 4 have provided evidence of differences between the different cues, they reveal little about the origin of these differences. Experiments 5 and 6 were designed to shed light on two possible influences. Experiment 5 establishes the contribution of eye movements to the longer response times for gaze cues, while Experiment 6 examines the visibility of the different cues in extrafoveal vision.

## Experiment 5

Experiments 1 to 4 provide consistent rankings between various cues of direction, suggesting that the cues with a clear outline (pointing hands, rotated heads and arrows) can be responded to more quickly and provide more response interference. One possible reason that these cues can be responded to more easily is that their direction can be more easily perceived away from fixation. In such an explanation, cues that cannot be perceived easily first need to be fixated before a response can be made, which takes time. Experiment 5, therefore, investigates whether the four social cues from Experiment 1 differ in the eye movements that participants make in response to the cues. If differences between the cues are due to their visibility in extrafoveal vision, we expect participants to more often look at the FACE and EYES cues than at the HAND and HEAD cues.

### Methods

Twelve participants (9 female, aged between 18 and 35 years of age) took part in the experiment in return for candy or course credit.

Stimulus presentation was controlled by a standard PC running Experiment Builder (SR Research, Ontario Canada) under Windows 7. Stimuli were presented on a Viewsonic VX2268 WM flat screen. An Eyelink 1000 system (SR Research) was used to measure the movements of the participants’ right eye at a 1000Hz sampling rate. A chin-and-forehead rest was used to restrict head movements in the participants and control the viewing distance to the screen to 62cm. Responses were collected using two keys at the bottom of a USB game-pad (Microsoft Sidewinder).

Experiment 5 applied the same stimuli, design, and procedure as in Experiment 1. Stimulus size and distance to the center of the display (in degrees of visual angle) were matched to those in Experiment 1 (adjusting for the change in viewing distance). Before starting the experiment, the eye tracker was calibrated using the standard nine-point calibration procedure, resulting in a reported 0.25–0.5 degrees average accuracy (SR Research).

The raw eye movement signal was parsed into fixations and saccades using the default settings of the Eyelink 1000 system. To analyze the eye movement patterns, a regions of interest analysis was performed for rectangular regions of interests around the two cues and the fixation point.

### Results

**Fig. 8:**
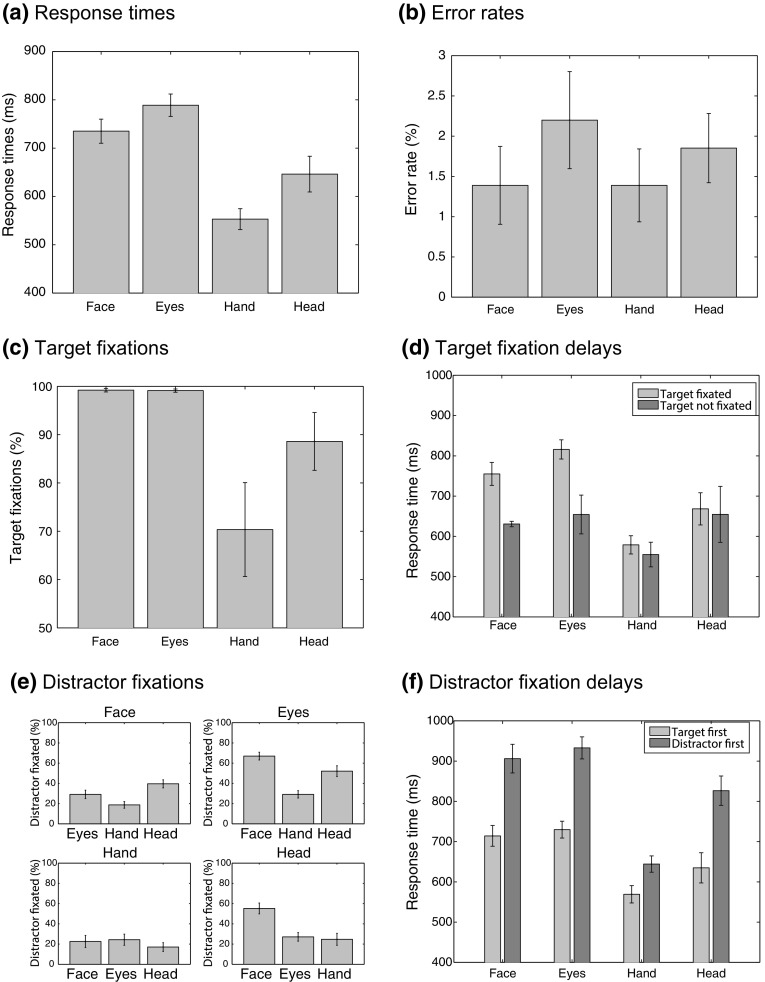
Results from Experiment 5, in which eye movements were recorded while participants performed the cue discrimination task in a setup identical to Experiment 1. **a** Response times pooled across congruent, incongruent and neutral conditions for the four cue types. **b** Error rates pooled across congruent and incongruent conditions. **c** Percentage of trials in which the target cue was fixated. **d** Response times for trials with and without fixations on the target. Note that some of these data are based on a subset of the participants (some participants always fixated the target, and one never fixated the hand). **e** Percentage of trials in which the distractor was fixated. **f** Response times for trials in which the cue was fixated first versus those in which the target was fixated first (data of the participant who never fixated the hand excluded). *Error bars* show the standard error of the mean across participants with observations (color figure online)

Figure 8 provides an overview of the results of Experiment 5. Because comparisons between congruent and incongruent conditions did not reveal clear distraction effects on response times and error rates (as in Experiment 1), pooled measures were used to compare the cues based on all (correct) response times and errors across congruent, incongruent, and neutral conditions, yielding a measure based on a large number of trials per participant (data split for congruent, incongruent and control conditions can be found in the online materials). In agreement with the no-distractor condition of Experiment 1, the pooled response times (Fig. 8a) showed fastest responses to HAND and HEAD cues and slower responses to FACE and EYES cues (main effect of cue type: *F*(3,33) = 33.2, *p* < 0.001, $\eta _\mathrm{p}$ = 0.75; Paired comparisons, applying a criterion of *p* = 0.05/6 = 0.0083, showed significant differences between FACE and HAND, *t*(11) = 8.17, *p* < 0.001, FACE and HEAD, *t*(11) = 3.23, *p* = 0.008, EYES and HAND, *t*(11) = 13.7, *p* < 0.001, and EYES and HEAD, *t*(11) = 4.78, *p* < 0.001). Error rates (Fig. 8b) were low and did not differ significantly across cues [*F*(3,33) = 0.79, *p* = 0.51, $\eta _\mathrm{p}$ = 0.067].

The eye movement data showed that FACE and EYES cues were fixated on almost every trial (Fig. 8c), but that HAND and HEAD cues were looked at less often [main effect of cue: *F*(1.48, 16.3) = 7.18, *p* = 0.009, $\eta _\mathrm{p}$ = 0.395, but paired comparisons between cues did not survive Bonferroni correction], suggesting that saccades to the target cue may have contributed to the slower responses for the FACE and EYES cues. To examine this possibility, Fig. 8c plots the average response times for trials with a fixation on the target and those without a fixation on the target. These data suggest that responses were slower without a fixation on the target for FACE and EYES cues, but not for HAND and HEAD cues. Note, however, that these data need to be interpreted with great caution, since averages in this plot tend to be based on a subset of the participants (e.g., most participants always fixated the FACE cue, and one participant never fixated the HAND cue).

Another possible reason for slow responses is that distractors are fixated before the target. Fixations on distractors varied across target–distractor combinations (Fig. 8d), revealing a complex pattern of results. Generally, these results suggest that distractor fixations occurred more often when the two cues were both provided by a human head. The majority of these trials (70 % or more) involve trials in which the distractor is fixated before the target (data not shown). To examine whether such distractor fixations influence responses times, Fig. 8f compares response times for trials in which the distractor was fixated first against trials in which the target was fixated first, separated across the different target cues, but pooled across distractors (data of one participant not included, who never fixated the hand). These data suggest that distractor fixations increase response times, but that this effect is weaker for the HAND cues [interaction between fixation sequence and target cue: *F*(3,30) = 9.23, *p* < 0.001, $\eta _\mathrm{p}$ = 0.48]. Paired comparisons between distractor-first and target-first trials show significant effects of fixating the target for all cue types (*p* < 0.001 for each comparison).

### Discussion

Experiment 5, in which eye movements were recorded while participants performed the cue direction discrimination task of Experiment 1, suggests that eye movements towards the cue may explain why the gaze cues (FACE and EYES) were responded to more slowly than the other cues (HAND and HEAD). Participants tended to fixate the gaze cues more often, and responses with a fixation on the cue tended to be slower than those in which the cue was not fixated (but these latter data need to be taken with caution, because some participants always fixated the gaze cues). The eye movements revealed another possible reason why gaze cues were responded to more slowly. If the distractor was fixated before the target, response times were slower, and fixations on the distractor more often occurred when the two cues were both provided by a human head.

The eye movement data, however, do not show why the gaze cues were fixated more often. It may be that their direction is more difficult to perceive from extrafoveal vision. Experiment 6, therefore, examines whether responses to the two gaze cues (FACE and EYES) are less accurate when participants are required to remain fixated on the fixation point during the task.

## Experiment 6

Experiment 5 showed that participants more often looked at the EYES and FACE cues, compared to the HEAD and HAND cues, in agreement with poor visibility of the cue’s direction in extrafoveal vision. To test this extrafoveal visibility directly, Experiment 6 forced participants to maintain fixation, and measured response times and accuracy when reporting the direction of the extrafoveally presented cues. If differences in extrafoveal visibility underlie the response time differences in Experiments 1, 3 and 4, we expect accuracy to be poorer to FACE and EYES cues, compared to HAND and HEAD cues, and response times to be longer.

### Methods

**Fig. 9:**
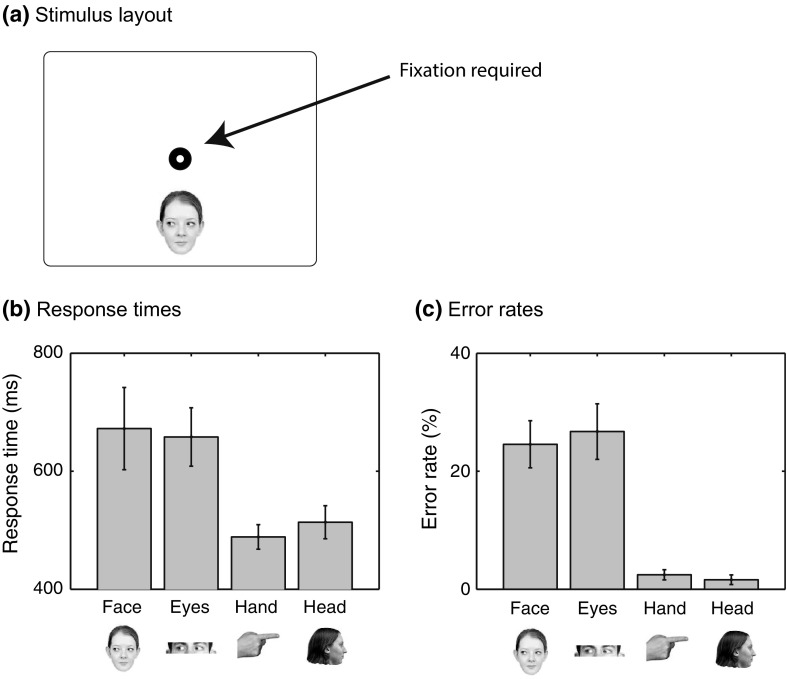
Stimulus layout and results from Experiment 6 in which participants were asked to report the direction of the cues while maintaining fixation on the fixation symbol. **a** Stimulus layout. Cues (as shown below the data plots in **b** and **c**), were presented above and below fixation (at the same distance as in previous experiments) without a distractor. Participants were asked to maintain fixation on the fixation symbol and report the direction of the cue by pressing one of two keys on a game-pad. **b** Response times (correct responses without fixations on the cue). **c** Error rates (without fixations on the cue). *Error bars* show the standard error of the mean across participants

Eight participants (4 female, aged between 18 and 39 years) took part in Experiment 6 in return for candy. The same apparatus as in Experiment 5 was used, where the eye tracker was used to confirm fixation on the fixation point, and to allow for the removal of trials in which fixation was not maintained. Participants performed 192 trials in which they were asked to report the direction of the cue (FACE, EYES, HAND, HEAD; same size as in previous experiments), presented above or below fixation (equal numbers of trials; at same distance as in the previous experiments, see Fig. 9a) and gazing or pointing left or right (equal number of trials). Trials were presented in a random order, and targets were always presented without a distractor, so that it was clear what stimulus to report without the need of an instruction at the start of the trial. Prior to the experiment, the eye tracker was calibrated using the standard nine-point calibration procedure. Participants were instructed to remain fixated on the fixation point, and to use the two keys at the bottom of a game-pad to indicate the direction of the cue. They were asked to respond as quickly and accurately as possible. After each 15 trials a short break was introduced. A regions of interest analysis with the fixation point and the target as regions of interest was conducted. Any trial with a fixation on the target was excluded from the analysis.

### Results

Response times and error rates showed the same pattern of results (Fig. 9b, c), which was also in agreement with findings of the previous experiments. Response times differed significantly across cues [*F*(3,21) = 13.8, *p* = 0.005, $\eta _\mathrm{p}$ = 0.66]. Bonferroni-corrected *t* tests (criterion for significance adjusted to 0.0083) showed significantly slower response times between EYES and HAND cues [*t*(7) = 5.15, *p* = 0.001] and between EYES and HEAD cues [*t*(7) = 5.27, *p* = 0.001]. Error rates also significantly differed across cues [*F*(1.62,11.4) = 23.9, *p* < 0.001, $\eta _\mathrm{p}$ = 0.77). Bonferroni-corrected t tests showed significant differences between all cues (*p* values <0.001), except between EYES and FACE cues, and between HAND and HEAD cues.

These results suggest that the faster response times to the HAND and HEAD cues (Experiments 1 and 3) and the stronger interference from these cues when measured using mouse trajectories (Experiment 4) are due to how much time is needed to determine the direction of the cue using covert attention, and how accurately the direction of the cue can be determined without fixating it. While it cannot be excluded that some of the response time differences are due to differences in how fast covert attention can be shifted to the cue, there is no a priori reason to believe that such attention shifts are faster for HEAD and HAND cues than for EYES and FACE cues.

## General discussion

While the majority of studies investigating social attention have focused on cues presented at fixation (Driver et al., 1999; Friesen & Kingstone, 2003a; Frischen et al., 2007a; Kuhn & Kingstone, 2009; Langton et al., 2000; Nummenmaa & Hietanen, 2006; Shepherd, 2010), researchers have also started to look into the effects of social cues away from fixation (Burton et al., 2009; Langton & Bruce, 2000; Nummenmaa & Hietanen, 2009; Yokoyama, Sakai, Noguchi & Kita, 2014). Studying the influence of extrafoveal social cues is important, because in day-to-day viewing, it is not uncommon to perceive social cues in the periphery first, before making an eye movement to it (Birmingham, Bischof & Kingstone, 2008, 2009). For example, when entering a room, observers may first look at a region of the room that is not occupied by a human face or body. Studies in which the fixation point preceding the presentation of a natural image was placed outside the image, show that people tend to make an eye movement to the center of the image or display, independent of where people appeared in the scene (Bindemann, 2010; Bindemann, Scheepers, Ferguson & Burton, 2010; Hermens & Walker, 2015), and it is, therefore, likely that something similar occurs when entering a room. To make use of social cues provided by people in the room, faces and bodies need to be detected, and an eye movement programmed to these sections. Past studies of social cues presented in extrafoveal vision have predominantly studied situations in which eye movements to extrafoveal cues were prevented (Burton et al., 2009; Nummenmaa & Hietanen, 2009). Similar differences in the effectiveness of extrafoveally presented cues, however, were found when eye movements were allowed (Langton & Bruce, 2000). On the basis of these past studies using extrafoveal cues, we formulated the hypothesis that extrafoveally presented cues with a clear shape exert strongest influences on responses, while the biological relevance of the cue is of less importance, even when eye movements towards the cues are allowed. This hypothesis is confirmed by the present results, with fastest responses to cues with a distinct outline (pointing hands, rotated heads, and arrows), compared to cues without such a clear outline (eyes within a face, eyes in isolation, direction words). These results are independent of whether participants respond to the cues themselves or to cued objects (suggesting that cue shape influences both response selection and attention shifts, or that these processes are strongly linked). Cues that are responded to quickly as a target were also stronger distractors (when measured by mouse trajectories). These cues were also less often fixated (possibly because their direction could be perceived without making an eye movement), and were easier to respond to when no eye movements were allowed. These results are summarized in Table 1.

**Table 1:** Overview of the main findings

Experiment	Stimuli	Dependent measure	Result
1	FACE, EYES, HAND, HEAD	Response times to cues	Fastest responses to HANDs and HEADS, slower to FACEs and EYES
2	ONSET, ARROW, EYES, WORD	Response times to cues	Fastest responses to ONSETs, followed by ARROWs, EYES and WORDs
3	FACE, EYES, HAND, HEAD	Response times to cued letters	Fastest responses to letters cued by HANDs, HEADs, slower to FACEs and EYES
4	FACE, EYES, HAND, HEAD	Mouse trajectories	Strongest interference from HANDs, HEADs, followed by FACEs and EYES
5	FACE, EYES, HAND, HEAD	Eye movements	More saccades to FACEs, EYES, fewer to HEADS and HANDs
6	FACE, EYES, HAND, HEAD	Response times and error rates to cues in isolation. Eye movements not allowed	Faster responses and higher accuracy for HANDs and HEADs, followed by FACEs and EYES

Our results are mostly in line with previous findings. For example, by asking participants to identify the direction of gaze of a backwardly masked face stimulus presented away from fixation, Yokoyama et al. (2014) showed that extrafoveally presented leftward and rightward gaze cannot be distinguished. This may explain the relatively weak effects of our gaze cues. Interestingly, Yokoyama et al. (2014) also found that when the task was to distinguish between averted and direct gaze, accuracy was high. It is unclear, however, how this latter result fits in our findings, as we only presented gaze cues with averted gaze. Our findings also agree with those from Langton and Bruce (2000), who used a similar interference paradigm, but instead used pairs of cues produced by a single actor. For example, photographs were used of actors looking up while pointing down, or actors looking down with an upward arrow painted on their shirt. As in the present study, both cues were presented away from fixation, and instructions before each block indicated which cue was the target and which cue the distractor. In agreement with the present results, Langton and Bruce’s (2000) found that targets with faster response times led to stronger interference when used as distractors. They also found stronger interference from pointing cues than from head orientation. Interestingly, they also found that the interference effects only occurred when the task was related to judging the direction of the cue. In Langton and Bruce's (2000) experiments, however, cues could sometimes be in the same vertical position (e.g., when the actor was pointing up and looking up or down, the pointing hand is next to the face), and the cues were not always identical for the different directions (e.g., the arm for the pointing up cue had a different shape than the arm that was pointing downwards), and our experiment may, therefore, provide better controlled stimuli. The weaker cueing by head orientations in Langton and Bruce (2000) is in line with our interpretation that the shape of the outline of the cue is important. The upward and downward postures of the head are less distinct than the leftward and rightward orientations that we used. Our results agree only in part with those by Nummenmaa and Hietanen (2009), who found similar cueing and distraction effects from (cartoon) gaze and arrow cues. It is unclear at this stage what caused these differences in results. The comparison of Experiments 1 and 3 suggests that task is unlikely to be the cause. Differences in the size of the stimuli is also unlikely to be a cause, as variations of stimulus size (Burton et al., 2009) and stimulus saliency (Nummenmaa & Hietanen, 2009) have not been found to make a difference. Our experiments also showed that neither cartoon nor photographs of gaze cues were easily responded to, and this distinction can, therefore, not explain the discrepancy either.

These results may have strong implications for theories of social attention. Our findings suggest that the strong cueing by gaze cues found in a broad range of studies (Driver et al., 1999; Friesen & Kingstone, 2003a; Frischen et al., 2007a; Kuhn & Kingstone, 2009; Langton et al., 2000; Nummenmaa & Hietanen, 2006) may be restricted to cues presented at fixation, and may not extend to cues (initially) presented away from fixation. In day-to-day viewing, immediately fixating someone’s face may be an uncommon situation (Bindemann 2010; Bindemann et al., 2010; Hermens & Walker, in press, for natural scenes, and Macdonald and Tatler, 2013 for direct interaction), suggesting that people may avoid frequently gazing at each other. Therefore the strong cueing by eye-gaze cues may be restricted to lab situations. Our data suggest that in everyday viewing gaze cues provided by rotated heads, body direction, and pointing gestures may be of higher importance than the eye region of the face. This may also explain why studies with cues in natural scenes found cueing effects for these types of cues (Fletcher-Watson, Findlay, Leekam & Benson, 2008; Gregory et al., 2015; Hermens & Walker, in press; Kuhn et al., 2009; Zwickel & Võ, 2010). Studies showing eye-gaze cueing in natural scenes placed the cues at fixation (Nummenmaa, Hyönä & Hietanen, 2009). The one exception appears to be a study by Hutton and Nolte (2011) who found longer dwell times for an object looked at by an actor not only having their head turned towards the object, but also their eyes. However, it needs to be determined how dwell times relate to the more commonly used measures of response times to cues or cued objects.

In our experiments, participants first had to locate the target after which they needed to respond to the cue’s direction. This situation resembles those of past studies using extrafoveal cues, and in particular that of Nummenmaa and Hietanen (2009), where participants had to shift their focus on the cue presented orthogonal to the cue’s direction. Because the direction of this shift of attention is orthogonal to the direction of the attention shift associated with the cue, no interference between the two is to be expected. In contrast, Langton and Bruce (2000) used cues directed along the axis along which the cues were presented, possibly leading to stimulus–response congruency effects. However, these should also cancel out when averaged across the positions of the stimuli.

One may question to which extent our results reflect automatic effects of the cues’ direction. Because participants were asked to respond to the direction of the cues, differences in response times to the stimuli as response targets may reflect voluntary effects. In contrast, influences of the distracting cues may provide a measure of automatic effects: even while participants had to ignore these cues, some of the distracting cues influenced response times (Experiment 2) and mouse trajectories (Experiment 4). However, because the overall task of participants is to respond to the direction of stimuli that all indicate a direction, one may also argue that some of the effects of the distractors could be voluntary, particularly if these stimuli were response targets themselves before being distractors. Likewise, some component of the response times to the stimuli as response targets may reflect automatic effects (faster responses, simply because the stimulus automatically generates a sense of direction). To disentangle automatic and voluntary effects, future studies could start with using distractors that are never response targets. If these distractors still influence responses to a target stimulus, then this would suggest that their associated response is not due a stimulus–response coupling.

Our experiments in which we recorded eye movements and in which participants were not allowed to look at the targets, suggest that gaze cues were responded to slowly as targets and were weak distractors during mouse tracking, because their direction was difficult to perceive in extrafoveal vision. This result converges with past findings on visual crowding (Levi, 2008), where features of peripheral objects are more difficult to report when these are presented with flankers. The role of crowding in reporting facial features of peripherally presented faces was demonstrated by Martelli, Majaj and Pelli (2005), who showed that despite a face familiarity effect, the shape of the mouth within a peripherally presented face could only be reported accurately when the remaining facial features were moved away from the mouth. Crowding may provide a likely explanation for our findings, but it may not explain why there was little difference between the full face gaze cues and the presentation of the eyes only. Likewise, crowding cannot explain why the cartoon gaze cue did not provide stronger cueing in Experiment 2. Therefore, crowding may only be part of the explanation of why gaze cues are not as efficient in extrafoveal vision as at fixation. While our experiments provide a first indication of the importance of distinguishable features in peripheral vision for attentional cueing, more detailed experiments, with a broader range of stimuli, will be needed to exactly identify what can make a directional cue a strong cue.

## Conclusion

At fixation, perceiving someone’s averted gaze strongly influences attention and response preparation in the observer. In contrast, the current study shows that extrafoveal gaze cues only exert weak influences, even when eye movements towards these cues are allowed (mimicking the situation in natural vision). Instead, our results indicate that extrafoveally presented cues with a clear outline, such as pointing hands and arrows, have a much stronger effect on response selection and visual attention. These results are relevant for understanding social attention in a natural context.
